# Applying the Risk of Bias Tool in a Systematic Review of Combination Long-Acting Beta-Agonists and Inhaled Corticosteroids for Persistent Asthma

**DOI:** 10.1371/journal.pone.0017242

**Published:** 2011-02-24

**Authors:** Lisa Hartling, Kenneth Bond, Ben Vandermeer, Jennifer Seida, Donna M. Dryden, Brian H. Rowe

**Affiliations:** 1 Alberta Research Centre for Health Evidence, Department of Pediatrics, University of Alberta, Edmonton, Alberta, Canada; 2 Department of Emergency Medicine, University of Alberta, Edmonton, Alberta, Canada; 3 School of Public Health, University of Alberta, Edmonton, Alberta, Canada; University of Modena and Reggio Emilia, Italy

## Abstract

**Background:**

The Risk of Bias (RoB) tool is used to assess internal validity of randomized controlled trials (RCTs). Our objectives were to: 1) evaluate inter-rater agreement of the RoB tool; 2) determine the time to access supplemental study information; 3) compare the RoB tool with the Jadad scale and Schulz allocation concealment (AC); and 4) examine the relationship between RoB and effect estimates.

**Methods:**

We conducted a systematic review of long-acting beta agonists (LABA) combined with inhaled corticosteroids (ICS) for adults with persistent asthma. Two reviewers independently assessed 107 trials using RoB, Jadad, and AC. One reviewer searched for study protocols. We assessed inter-rater agreement using weighted Kappa (κ) and the correlation between tools using Kendall's Tau (τ). Mean differences in effect sizes for RCTs with different RoB were calculated using inverse variance method and random effects model.

**Results:**

Trials had good Jadad scores (median 4, IQR 3-4); however, 85% had unclear AC and 87% high RoB. The factor that most influenced RoB was the potential inappropriate influence of study sponsors (95% industry funded). Agreement on RoB domains was fair (κ = 0.40) to almost perfect (κ = 0.86), and moderate for overall RoB (κ = 0.41). Median time to complete RoB assessments was 21 minutes (IQR 14-27) and 12 minutes (IQR 9-16) to search for protocols. Protocols were identified for 5/42 studies (12%); in 3 cases the assessment of selective outcome reporting changed. There was low correlation between overall RoB vs. Jadad (**τ** = 0.04, p = 0.3) and AC (**τ** = −0.02, p = 0.7). Analyses comparing effect estimates and risk showed no important patterns.

**Conclusions:**

Inter-rater agreement on RoB assessments was better than previously reported suggesting that review-specific guidelines are important. The correlation between RoB and Jadad was low suggesting measurement of different constructs (risk of bias vs. quality of reporting). The extensive involvement of the pharmaceutical industry in this LABA/ICS research should raise concerns about potential overestimates of treatment effects.

## Introduction

Assessing the methodological quality, or risk of bias, of studies included in a systematic review (SR) is a key methodological step and serves to identify the strengths and limitations of individual studies; investigate, and potentially explain, heterogeneity in findings across different studies included in a SR; and, contribute to grading the quality of a body of evidence for a given question. There are numerous tools to assess methodological quality of primary studies; however, few have undergone extensive inter-rater reliability or validity testing. It is unknown whether, or to what extent, quality assessments based on existing tools differentiate studies that may yield biased results either by over or underestimating treatment effects. Such information is critical for decision-making in order to gain an accurate assessment of the potential benefits (or harms) of a given intervention.

In 2008, The Cochrane Collaboration released a new tool to assess risk of bias in randomized controlled trials (RCTs) [1]. The Risk of Bias (RoB) tool was developed through an extensive process in order to improve on other tools used for quality assessment [1]. The RoB tool comprises six domains: sequence generation, allocation concealment, blinding, incomplete outcome data, selective outcome reporting, and “other sources of bias.” These domains were chosen based on empirical evidence demonstrating potential for bias or exaggeration of treatment effects. For instance, numerous meta-epidemiological studies have demonstrated that RCTs with inadequate or unclear allocation concealment can overestimate treatment effects by 18% on average [2]. Table 1 identifies common sources of bias and the relevant domains of the RoB tool that assess each bias. Further, the tool was intended to assess the validity of results based on the features associated with the design and conduct of the study, rather than reporting.

**Table 1:** A classification scheme for bias (based on Table 8.4.1 in Cochrane Handbook for Systematic Reviews of Interventions [1]).

Type of bias	Description	Relevant domains in the Risk of Bias tool
Selection bias	Systematic differences between the baseline characteristics of the groups.	Sequence generationAllocation concealment
Performance bias	Systematic differences between the groups in the care that is provided, or in exposure to factors other than the interventions of interest.	BlindingOther sources of bias
Attrition bias	Systematic differences between groups in withdrawals from the study.	Incomplete outcome dataBlinding
Detection bias	Systematic differences between groups in how outcomes are measured.	BlindingOther sources of bias
Reporting bias	Systematic differences between reported and unreported findings.	Selective outcome reporting

Initial research examining the RoB tool showed that inter-rater agreement ranged from slight (κ = 0.13) to substantial (κ = 0.74) across the different domains, with overall risk of bias assessment being fair (κ = 0.27) [3]. The authors also provided preliminary data showing validation of the RoB tool: studies at high or unclear risk of bias had significantly greater treatment effects (effect size = 0.52) than those at low risk of bias (effect size = 0.23). The authors made recommendations for future research including: evaluating the tool within the context of a SR with pilot testing and established decision rules; quantifying time requirements when all main outcomes are included in the assessment; examining time requirements and impact on risk of bias assessments when searching for study protocols or additional study information; and, using a meta-epidemiological approach to assess validity in order to minimize confounding due to intervention and design.

Building on this previous research, we applied the RoB tool to a large SR examining combination long-acting beta-agonists (LABA) and inhaled corticosteroids (ICS) for maintenance therapy of persistent asthma [4]. We sought to: 1) assess inter-rater agreement of the RoB tool following rigorous pilot testing and review-specific decision rules; 2) assess the time to access supplemental study information and the impact of additional information on risk of bias assessments; 3) compare the RoB tool with two common approaches to quality assessment in SRs (Jadad scale [5] and allocation concealment [6]); and, 4) examine the relationship between risk of bias and effect estimates.

## Methods

### Study sample

The study sample was 107 RCTs that were included in a SR of combination LABA/ICS for maintenance therapy in persistent asthma. The median year of publication of these RCTs was 2004 (interquartile range [IQR] 2001, 2006). The methods of the SR, reported in detail elsewhere [4], are briefly described here. A comprehensive search of electronic databases and grey literature were performed to avoid publication and selection bias. Studies were included if they were RCTs involving the use of ICS/LABA combination agents in the treatment of chronic asthma.

As part of the SR, all RCTs were assessed for methodological quality independently by two reviewers using the Jadad scale [5] and allocation concealment [6]. Jadad is a 5-point scale comprised of items related to sequence generation, blinding, and withdrawals. It is the most widely used and frequently cited quality assessment instrument [7]. Allocation concealment has historically been used alone or in conjunction with the Jadad scale and is rated as adequate, inadequate, or unclear. Consensus on quality assessments were made through discussion between the two reviewers or adjudicated by a third reviewer. Data on effect estimates from individual studies were extracted as part of the SR: data were extracted by one reviewer and checked for accuracy and completeness by a second reviewer. All data were checked by the statistician during analysis.

### Risk of bias assessments

We conducted pilot testing of the RoB tool among the team of reviewers who would complete all risk of bias assessments. We relied primarily on guidelines for application of the tool developed by The Cochrane Collaboration [1]. For the “other sources of bias” domain, we regularly looked for baseline imbalances between study groups that could have biased the results or that were not accounted for; inappropriate influence of funders that could have biased the results; and early stopping for benefit. In addition, we developed several decision rules to address nuances specific to this SR, such as how to assess potential bias due to influence of funders (Appendix S1).

Each study was assessed independently by two reviewers and any discrepancies were resolved through discussion. The tool was applied for three outcome categories: pulmonary function tests, asthma control, and quality of life. For a sample of studies (40%), one reviewer searched for supplemental information (i.e., trial protocols) using a pre-defined protocol. This included searching online trial registries (www.who.int/trialsearch, www.clinicalstudyresults.org, www.controlled-trials.com/mrct) and performing a Google search using the name of the corresponding author, title, and key words. For each study, reviewers documented the time required to independently complete their RoB assessment, complete consensus, and search for additional study information.

### Analysis

Inter-rater agreement was calculated using weighted kappa (κ) statistics for each domain and for an overall RoB assessment. Agreement was categorized based on published norms: poor (0.00), slight (0.01–0.20), fair (0.21–0.40), moderate (0.41–0.60), substantial (0.61–0.80), and almost perfect (0.81–1.00) [8]. Correlations between the RoB tool and the Jadad scale and allocation concealment were calculated using Kendall's tau (τ). For each risk of bias domain and for overall RoB, we compared effect estimates for studies at high or unclear risk of bias vs. low risk of bias. We calculated mean differences in effect size using an inverse variance method and random effects model. A priori we planned to compare effect estimates for pulmonary function tests (i.e., FEV_1_), asthma control (i.e., symptom-free days), and quality of life. Due to insufficient quality of life data, we were unable to make meaningful comparisons.

## Results

### Overall methodological quality and risk of bias

The median Jadad score across the 107 RCTs was 4 on a five-point scale (IQR 3, 4); scores greater or equal to 3 are considered good methodological quality [9]; [10]. Allocation concealment was unclear in 91 (85%) and adequate in 16 (15%) studies. Ninety-three trials (87%) were at high risk of bias and 14 (13%) at unclear risk of bias. No studies were assessed as low risk of bias. The factor that was most influential for risk of bias was the potential for inappropriate influence of the study sponsor. Overall 95% of these LABA/ICS trials were funded by the pharmaceutical industry. In 35 studies (41%), the first or last author was affiliated with industry. Further, in 84 studies (79%), one of the authors was an employee of the pharmaceutical industry or the document was an industry report. We recalculated risk of bias without the funding component and found that 38 (36%) were high, 66 (62%) were unclear, and 3 (3%) were low risk of bias.

### Inter-rater agreement for risk of bias assessments

Inter-rater agreement (Table 2) varied across RoB domains. For example, the agreement was fair (0.40) for selective outcome reporting and almost perfect (0.86) for sequence generation. Inter-rater agreement for the majority of domains and overall risk of bias was moderate (κ = 0.41–0.60). Table 2 also compares the inter-rater agreement to that found in a previous study demonstrating improvement in all but one domain [3].

**Table 2:** Inter-rater agreement for individual domains and overall risk of bias.

Domain	Weighted kappa(95% CI)	Interpretation	Previous research (3)
Sequence generation	0.86 (0.74, 0.98)	Almost perfect	Substantial
Allocation concealment	0.54 (0.29, 0.79)	Moderate	Moderate
Blinding	0.62 (0.46, 0.79)	Substantial	Fair
Incomplete data	0.44 (0.27, 0.62)	Moderate	Fair
Selective reporting	0.40 (0.14, 0.67)	Fair	Slight
Other sources of bias	0.52 (0.33, 0.72)	Moderate	Fair
Overall risk	0.41 (0.19,0.62)	Moderate	Fair

### Time for risk of bias assessments and supplemental information search

The average time for one reviewer to complete the risk of bias assessment was 8.7 minutes per study (IQR 5.9, 11.4). The average time required for consensus between two reviewers was 1.5 minutes per study (IQR 0.5, 2.5). Overall time required for two reviewers to complete assessments and consensus was 20.5 minutes per study (IQR 14.4, 27.0). The average time spent searching for a study protocol or other supplemental study material was 11.7 minutes (IQR 9.1, 15.6). Supplemental study material was found for 5/42 (12%) of the trials. In 3/5 cases, assessment of selective outcome reporting changed, although the direction of changes was inconsistent: unclear to yes, yes to unclear, and unclear to no.

### Correlation of risk of bias and quality assessments

Tables 3 and 4 display the assessments for RoB compared to Jadad scores and allocation concealment, respectively. The correlations between overall risk of bias assessments and total Jadad score (τ = 0.04) and allocation concealment (τ = 0.02) were low. When the funding component was removed from the overall risk of bias assessments, the correlations remained low (τ = 0.17 vs. Jadad and 0.07 vs. allocation concealment).

**Table 3:** Assessment of risk of bias vs. Jadad scores (n = 107 trials; correlation, τ = 0.04).

	Jadad Scores	
	Good quality(score ≥3)	Poor quality(score <3)
**Risk of bias**		
Low	0	0
Unclear	13	1
High	82	11

**Table 4:** Assessment of risk of bias vs. allocation concealment (n = 107 trials; correlation, τ = 0.02).

	Allocation concealment		
	Adequate	Unclear	Inadequate
**Risk of bias**			
Low	0	0	0
Unclear	3	11	0
High	13	80	0

### Association between risk of bias and effect estimates

**Figure 1:**
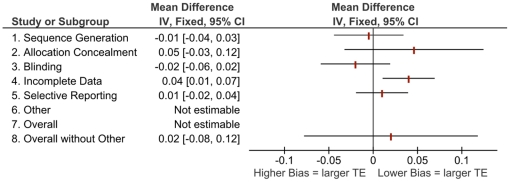
Mean differences in effect estimates for FEV_1_ across domains and overall risk of bias (with and without “other” sources of bias), high/unclear vs. low risk of bias (Note: TE = treatment effect; FEV_1_ = forced expiratory volume in 1 second. Components were not estimable when all studies were rated the same [i.e., all high/unclear or all low risk of bias]).

**Figure 2 pone-0017242-g002:**
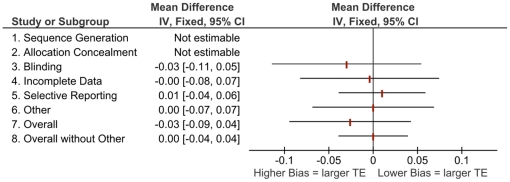
Mean differences in effect estimates for FEV_1_ across domains and overall risk of bias (with and without “other” sources of bias), high vs. low/unclear risk of bias (Note: treatment effect; FEV_1_  =  forced expiratory volume in 1 second. Components were not estimable when all studies were rated the same [i.e., all high or all low/unclear risk of bias]).

**Figure 3 pone-0017242-g003:**
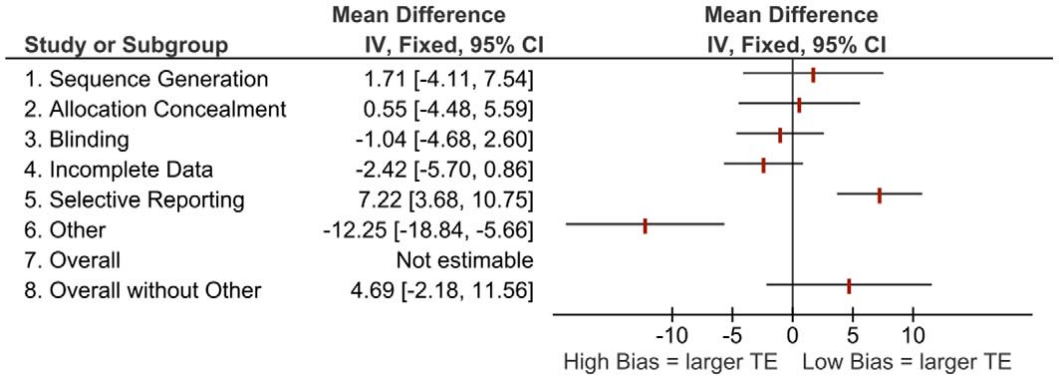
Mean differences in effect estimates for symptom-free days across domains and overall risk of bias (with and without “other” sources of bias), high/unclear vs. low risk of bias. [Note: TE  =  treatment effect]).

**Figure 4:**
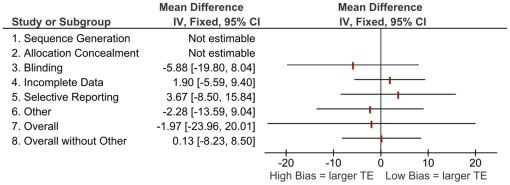
Mean differences in effect estimates for symptom-free days across domains and overall risk of bias (with and without “other” sources of bias), high vs. low/unclear risk of bias. (Note: TE  =  treatment effect. Components were not estimable when all studies were rated the same [i.e., all high or all low/unclear risk of bias]).

Figures 1 to [Fig pone-0017242-g002] [Fig pone-0017242-g003] 4 show the differences in effect estimates for studies at different risk of bias for each of the domains in the RoB tool as well as overall risk of bias, both with and without “other” sources of bias. Figures 1 and 3 compare high or unclear vs. low risk of bias, while Figures 2 and 4 compare high vs. unclear or low risk of bias. There were few notable differences observed which may be due to the homogeneity of study results in this review and small differences in effects within the original meta-analyses [4]. The one difference observed that may be of clinical importance is larger treatment effects for trials at high or unclear risk compared to those at low risk with a difference of 12 symptom-free days (Figure 3, other sources of bias).

## Discussion

This study examined the reliability of the RoB tool and compared this tool with other quality assessment tools currently employed in SRs. We demonstrated improved reliability for risk of bias assessments using the new RoB tool compared to previous research [3]. This may be due to the fact that this study was based on RCTs included in a real SR hence there was more consistency across trials with respect to a number of factors including populations, interventions, control interventions, outcomes, study design, and reporting. In contrast, the previous research was based on a sample of diverse pediatric trials that covered numerous interventions and conditions [3]. The improved reliability may also have stemmed from rigorous pilot testing, training of reviewers, increased familiarity with the RoB tool, and/or the set of decision rules specific to the SR.

The overall time required to complete the risk of bias assessments in this study was consistent with time reported in previous research [3]. In the present study, however, risk of bias was assessed for three clinically important outcome categories for the review, whereas the previous study assessed risk of bias for only one outcome. This suggests that time required by outcome may have decreased.

The time spent searching for supplemental study information, including the study protocol, added substantially (50%) to the total time for the risk of bias assessments. While the yield from these searches provided additional information in only 12% of studies, the additional information did result in differences in assessment of selective outcome reporting in 3 of the 5 cases. The potential for supplemental information to change assessments raises several items for consideration, especially as it pertains to selective outcome reporting. First, relying on the published or final trial report may not result in accurate assessment of selective outcome reporting. Second, since a substantial amount of time is required to systematically search for additional information, based on this study, the yield may not be worth the resource investment. We conducted a comprehensive search of various sources for supplemental study information and believe that the low yield was not due to insufficient searching but to a lack of availability of trial protocols and the inherent difficulties in searches of the grey literature. Since the median year of publication for our trials was 2004, recent initiatives such as universal trial registration and the Standard Protocol Items for Randomized Trials (SPIRIT) initiative may improve this in the future [11]; [12].

The low correlation between overall risk of bias assessments and total Jadad score was confirmed in this study. This may reflect the different domains included across the tools; however, the fact that different tools derive such divergent overall estimates of the quality or risk of bias of a body of literature is troublesome, particularly for decision-makers. Based on the Jadad scale, this sample of trials would be considered of good methodological quality. The RoB assessments, however, showed that the vast majority of studies were at high risk of bias and have the potential to overestimate treatment effects. The methodology for assessing the quality of studies in a SR continues to be an issue on ongoing debate. Previous research has identified inherent problems with the use of summary scores from quality scales, and different scales have been shown to lead to discordant results [13]. Many scales such as the Jadad also place undue emphasis on the reporting, rather than the conduct, of trials. Taken together, these limitations may suggest that the RoB tool is a more favorable approach for assessing the internal validity of studies.

The factor that was most influential for risk of bias was the potential for inappropriate influence of study sponsors. We included this variable within the “other sources of bias” domain; however, users of the RoB tool may consider examining this variable separately due to the complex nature of funding source and its influence on the design, conduct, and reporting of trials. Further, we developed our own guidelines to determine whether there was potential bias due to inappropriate influence of the study sponsor. Clear and consistent guidelines are needed for other users of the RoB tool. The majority of reported LABA/ICS trials for persistent asthma have pharmaceutical industry sponsorship or include industry employees as authors on the publications. There is substantial literature supporting the association between pharmaceutical funding and results favouring the sponsors' interests [14]. Efforts to ensure separation between the pharmaceutical industry and published research therefore appear warranted and urgently needed.

We were unable to demonstrate a clear association between risk of bias and effect estimates. This may be a result of several factors. First, there was considerable homogeneity in treatment effects and any observed differences were relatively small, therefore it may be unrealistic to expect differences across sub-groups. Second, there were few studies in the low risk of bias category which may have reduced the power to detect differences.

There were several limitations to this study. We explored our research questions within a SR in order to assess how the RoB tool performs in the context of a SR. The homogeneity among the studies and the relatively small sample of studies (n = 107) may have limited our ability to detect differences in effect estimates by risk of bias. The confidence intervals were wide and do not rule out the possibility of an association. Moreover, the majority of studies in this sample showed some potential for influence from the pharmaceutical industry. We did not follow-up with authors to confirm conflicts of interest or methods used to ensure separation between the pharmaceutical industry and researchers during the conduct, analysis, and reporting of trials; however, when we removed the funding item from our overall RoB assessments, we found that risk of bias remained high or unclear for the majority of studies. Finally, the RoB assessments were not conducted concurrently or by the same group of reviewers as the Jadad and allocation concealment assessments. This may have introduced some variability in the assessments and judgments made.

### Conclusion

The Risk of Bias tool is a new, Cochrane-recommended approach to assessing the internal validity of RCTs. This study demonstrates that the inter-rater reliability of the tool is enhanced with appropriate training, pilot-testing, and context-specific decision rules. Clear and consistent decision rules for the Risk of Bias tool regarding potential influence of the study sponsor are needed. The low correlation of risk of bias results with other approaches to assessing methodological quality suggests that the tool is measuring different constructs, and may be more appropriate to detect threats to internal validity. The risk of bias assessments did not differentiate effect estimates in this group of studies; however, the frequent and intimate involvement of the pharmaceutical industry in this body of literature should raise concerns about potential overestimates of treatment effects in favour of the sponsors' interests.

## Supporting Information

Appendix S1 **These decision rules are intended to supplement the criteria for assessing risk of bias as presented in the Cochrane Handbook for Systematic Reviews of Interventions.**
(DOC)
