# Supplementary material for: Applying the Risk of Bias Tool in a Systematic Review of Combination Long-Acting Beta-Agonists and Inhaled Corticosteroids for Persistent Asthma
Source: PLoS One. 2011 Feb 24;6(2):e17242. doi: 10.1371/journal.pone.0017242 (PMC3044729; doi:10.1371/journal.pone.0017242)
Supplement: Appendix S1 — These decision rules are intended to supplement the criteria for assessing risk of bias as presented in the Cochrane Handbook for Systematic Reviews of Interventions. (DOC) [file pone.0017242.s001.doc]

**Appendix S1.**

These decision rules are intended to supplement the criteria for assessing risk of bias as presented in the Cochrane Handbook for Systematic Reviews of Interventions.

**1 General Points**

- - If the article refers to other documents for additional information (e.g., online supplement), that information should be used to make the risk of bias assessments.

**2 Domain-specific points**

**Blinding:**

- This domain should be assessed for the following outcomes: pulmonary functions tests; asthma control; quality of life (QOL) (see details below under item 3). If the risk varies for different outcome measures within these outcome categories, assessment should be made based on the most subjective outcome measure, or the one most prone to bias.
- Patient assessed and reported outcomes are considered to be more subjective and more prone to bias (i.e., self-assessments).
- For this body of literature, assume that the term “double-dummy” implies that an appropriate method was used to blind the participants and study personnel. However, the same cannot be assumed if the term “double-blind” is used without some additional description of methods. The term ‘open label’ assumes that the participants and personnel were not blind to the intervention.

**Incomplete outcome data:**

- If authors claimed that an intention-to-treat analysis was performed, raters should confirm that all patients entered were accounted for in the analysis (i.e., do not assume that a true intention-to-treat analysis was done).
- If all patients were accounted for in the analysis (i.e., no drop-outs or censored analysis conducted), assume YES
- If the numbers and reasons for withdrawal/drop-out were described and comparable across groups (and ≤ approximately 10%), assume YES
- If there was greater than 10% drop-out, consider UNCLEAR or NO

**Selective outcome reporting:**

- Assess this outcome initially exclusive of the protocol (if available). Where available, also assess the outcome based on the protocol information.
- When assessing this domain without a protocol, compare the outcomes listed in the methods section to those reported on in the results section of the document for consistency and thoroughness (and ultimately risk of bias). Consider different outcomes, measurement tools, and timepoints.
- Forced vital capacity (FVC) would not be affected by treatment, so may be of more interest in describing the study population. If this variable is mentioned in the methods but not reported in the results, and all others are reported, assume YES. The same would hold for forced expiratory flow (FEF25-75).

**Other sources of bias:**

- Regularly assess:
  - whether there were baseline imbalances that could have biased the results (or were not accounted for)
  - inappropriate influence of funders that could have biased the results, and
  - early stopping for benefit.
- Document “other sources of bias” that are of concern.
- For influence of funders:
  - If sponsor is acknowledged and there is a clear statement regarding no involvement of sponsor in trial conduct or data management/analysis, answer YES for this item
  - If sponsor is acknowledged and noone from the sponsoring agency was an author, answer UNCLEAR for this item
  - If sponsor is acknowledged and someone from the sponsoring agency was an author, answer NO for this item
  - If there is no mention of funding source, answer UNCLEAR for this item.
  - There are a number of unpublished reports in this dataset that are industry-reported trials. These should be considered at high risk of bias.

**Overall risk of bias:**

- This item should be assessed using the Cochrane criteria.

**3 Categorization of outcome variables for assessment of ROB:**

**Pulmonary Function**

1. PEF AM

2. PEF PM

3. FEV1 % predicted

**Asthma Control**

4. Total no. exacerbations during study period

5. Time to 1st exacerbation

6. % pts with ≥1 exacerbation

7. No. severe exacerbations

8. No. mild exacerbations

9. SABA/reliever use (puffs/day)

10. Mean ICS dose

11. Asthma control questionnaire

12. Symptom-free days

13. Days with OC

14. Change in dose of ICS

**HRQoL**

15. AQLQ
